# Troponin May Lie: Recognizing an Atypical Case of Wellens Syndrome

**DOI:** 10.1002/ccr3.70440

**Published:** 2025-05-12

**Authors:** Juthipong Benjanuwattra, Cristian Castillo‐Rodriguez, Manisha Das, Shaheer Zulfiqar, Imran Arif

**Affiliations:** ^1^ Division of Cardiology University of Cincinnati Cincinnati Ohio USA; ^2^ Division of Internal Medicine Texas Tech University Health Sciences Center Lubbock Texas USA; ^3^ Division of Intervention Cardiology University of Cincinnati Cincinnati Ohio USA

**Keywords:** acute coronary syndrome, coronary artery disease, myocardial infarction, Wellens syndrome

## Abstract

Wellens syndrome is associated with a critical coronary artery stenosis and an impending extensive myocardial infarction. Despite having chest pain, both ECG and troponin can be misleading. It is crucial to recognize this syndrome to allow urgent revascularization. Failure to recognize and noninvasive stress tests were shown to be detrimental.

## 1 Introduction

Wellens syndrome, which was first described by Zwaan and Wellens in 1982, signifies a critical stenosis in the left anterior descending coronary artery (LAD) with characteristic ECG features [1]. The diagnosis can be overlooked or delayed as patients often present in a pain‐free state, and troponin is also not a reliable marker.

Recently, a concept of occlusion myocardial infarction (OMI) was proposed to shift a paradigm away from solely relying on the presence of persistent ST‐segment elevation as an indication for emergent reperfusion, as only about 40% of acute OMI fulfill the ST‐elevation myocardial infarction (STEMI) criteria and up to 30% of non‐STEMI are found to have OMI [2]. Several ECG patterns predictive of OMI, without persistent ST‐segment elevation, include De Winter T waves, left bundle branch block meeting Sgarbossa criteria, diffuse ST‐segment depression with ST‐segment elevation in aVR, and, unsurprisingly, Wellens syndrome [3].

Hereby we present a case of a male patient with intermittent chest pain and unremarkable troponin, with Wellens syndrome and severe multivessel disease. The case highlights the importance of clinical awareness of this condition to allow timely diagnosis and revascularization.

## 2 Case History/Examination

A 70‐year‐old man with a history of hypertension, hyperlipidemia, prior stroke, and frontotemporal dementia presented with intermittent chest pain for the past 3 days. Three days prior, he had sharp substernal pain that resolved spontaneously within 20 min. Physical examination was normal. ECG did not show any specific ST‐T abnormality. High‐sensitivity troponin (hs‐cTn, Abbott) was at 13 ng/L, which was below the 99th percentile of the upper reference level (20 ng/L), and delta 2‐h of 2 ng/L. Based on the current guideline [4], acute myocardial infarction (MI) was ruled out, and he was discharged.

**FIGURE 1:**
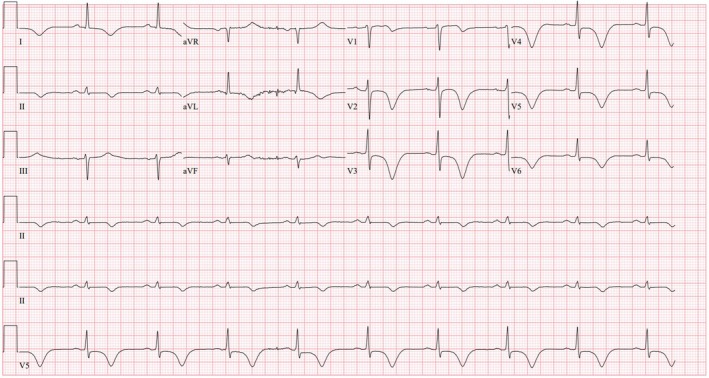
ECG showing deep symmetrical T wave inversion across the precordial, I, and aVL leads, associated with prolonged QT interval.

He then presented again with severe substernal pressure and dyspnea that lasted for 1 h. En route to the hospital, he was hypertensive to 200/100 mmHg. He received aspirin 325 mg. Upon arrival, he was normotensive and other vital signs were normal. Chest pain had resolved. Initial hs‐cTn was elevated but below the MI rule‐in value (27 ng/L) with a repeat hs‐cTn at 33 and 34 ng/L (delta 2‐ and 3‐h of 6 and 1 ng/L, respectively) (Table 1). ECG, which was obtained in a pain‐free state, showed sinus bradycardia with a prolonged QT interval and deeply inverted T waves across the precordial, I, II, and aVL leads (Figure 1). He was initially deemed to have demand ischemia associated with a hypertensive emergency. A noninvasive stress test was planned as a diagnostic test given that the patient had an intermediate risk with no known coronary disease [5]. However, several hours later, he developed a third episode of chest pain with blood pressure up to 200/100 mmHg. A heparin drip was started. Chest pain and hypertension were resolved with sublingual nitroglycerin.

**TABLE 1:** High‐sensitivity cardiac troponin levels at varying times following arrival to the emergency room.

Time	First presentation	Second presentation (ng/L)
Arrival	13 ng/L	27
2 h after arrival	15 ng/L	33
Delta 2 h	2 ng/L	6
5 h after arrival	n/a	34
Delta 3 h	n/a	1

## 3 Differential Diagnosis, Investigations, and Treatment

Cardiology was notified, and a decision was made to perform an urgent coronary angiogram because of a concern of Wellens syndrome, as suggested by the intermittent nature of chest pain and characteristic ECG findings.

**FIGURE 2:**
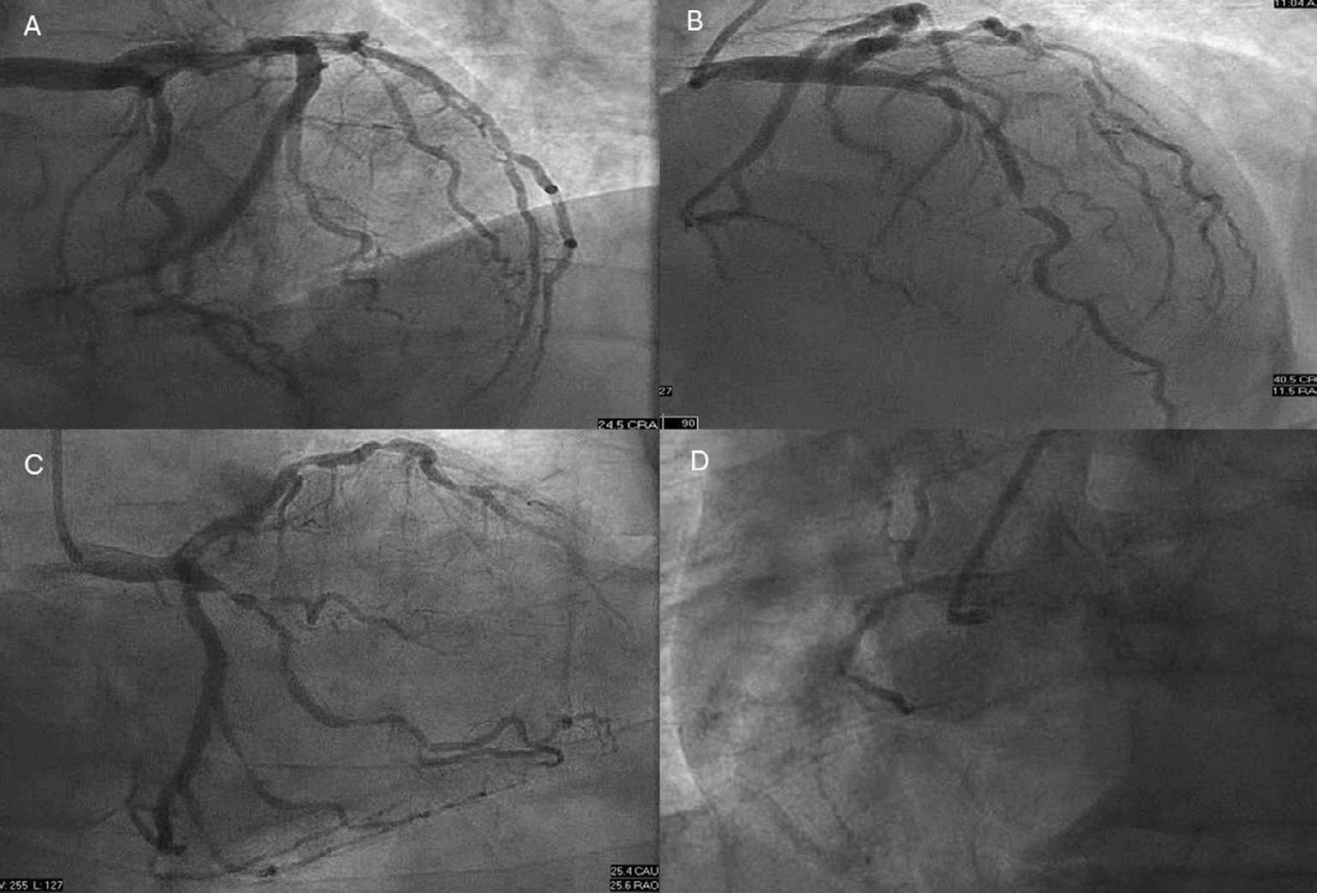
Coronary angiogram showing critical 99% stenosis in the mid left anterior descending artery, critical stenosis of the first diagonal branch, and severe 95% stenosis of the proximal first obtuse marginal and second obtuse marginal branches (A–C). Chronic total occlusion of the right coronary artery was shown (D).

Coronary angiogram revealed multivessel disease (Figure 2). There was a critical stenosis in the mid left anterior descending coronary artery (LAD) and the ostium of the first diagonal branch (D1), severe stenosis of the proximal segment of the first and second obtuse marginal (OM) branches, and chronic total occlusion of the RCA. Echocardiography, which was obtained after revascularization, showed normal function without wall motion abnormality.

## 4 Outcomes and Follow‐Up

**FIGURE 3:**
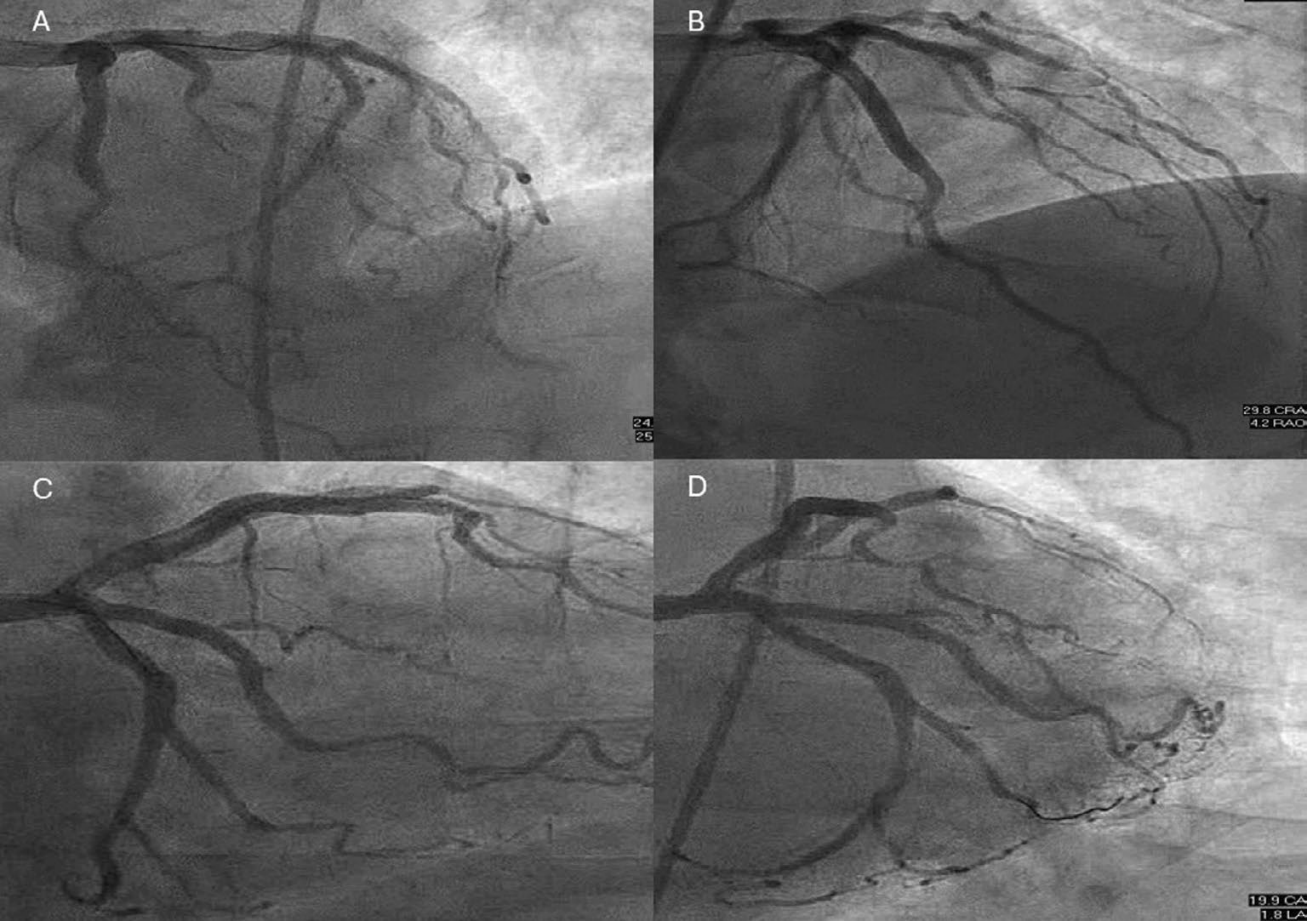
Coronary angiogram showing TIMI‐3 flow in the left anterior descending artery, first diagonal, and both obtuse marginal branches following coronary intervention.

Patient underwent percutaneous coronary intervention (PCI) to the LAD and D1 with drug‐eluting stents. Staged PCI was later performed to both OM branches (Figure 3). There was no periprocedural MI or cardiovascular event during follow‐up.

## 5 Discussion

Wellens syndrome is a condition with specific ECG patterns associated with critical stenosis of the LAD [1, 6]. The risk factors for Wellens syndrome include the traditional risk factors for coronary artery disease, such as hypertension, diabetes mellitus, dyslipidemia, obesity, smoking, and family history of premature coronary disease [7]. Interestingly, those with Wellens syndrome are less likely to have a personal history of coronary disease or previous PCI compared with non‐Wellens patients [7].

The ECG is characterized by biphasic T waves in leads V2–V3 (Type A) or primarily deeply inverted T waves in leads V2–V3 that can extend across all precordial leads (Type B) [1, 6]. The Type B pattern is much more common and represents approximately 75% of cases [8]. Other suggestive ECG features include absence of pathological precordial Q wave, preserved precordial R wave progression, and isoelectric or minimal ST‐segment elevation (< 1 mm) [9].

The finding of ST depression in multiple leads, particularly in eight or more leads, together with ST elevation in aVR and/or V1 is suggestive of severe multivessel or left main disease [10]. As in our case, there are widespread > 8 leads with T wave inversion, including lead I and aVL, with minimal ST elevation in V1, which is most likely explained by occlusion of LAD and OM branches. Another case report of Wellens syndrome with both LAD and proximal circumflex stenosis also exhibited T wave inversion in lead I and aVL [11].

The prevalence of Wellens syndrome in a cohort of patients with unstable angina was approximately 14% on the basis of a study conducted by de Zwaan et al. [8] over three decades ago. The proximal and mid LAD lesions were found to be culprits in 29% and 54% of patients, respectively. Multivessel disease was found in 22% of patients [8]. Among those who were not revascularized, approximately 30% of cases developed acute MI during follow‐up [6]. In the current era of hs‐cTn, the prevalence of Wellens syndrome has changed [7]. Based on a cohort of 2,127 patients with non ST‐elevation ACS from a culprit LAD, there were 200 patients (9.4%) fulfilling the ECG criteria for Wellens syndrome [7]. A relatively similar prevalence of 8.8% was reported from another retrospective cohort study [6]. The prevalence of multivessel disease did not differ between those with and without ECG characteristics of Wellens syndrome. However, based on this single center retrospective study, the prevalence of multivessel disease was almost four times higher than the previously reported prevalence of 22% [7]. The presence of the Wellens sign had a sensitivity of 24.6% and a specificity of 96.2% in predicting a culprit LAD lesion [6].

The characteristic feature of Wellens syndrome involves coronary artery stenosis with intermittent reperfusion and reocclusion that can develop over days to weeks, thus explaining the dynamic change of ECG findings and stuttering nature of chest pain [12]. Although not shown in our patient, clinicians must be aware that T wave may appear normal and upright during a period of reocclusion and active chest pain, which is described as “pseudonormalization.” Acute ST‐elevation MI can eventually develop if the artery remains occluded [12].

Patients typically present during a pain‐free state, with normal or slightly elevated cardiac biomarkers [9]. As in our patient, even hs‐cTn is falsely reassuring. Recognizing these ECG patterns and the nature of chest pain are crucial, as these patients require early coronary revascularization because of the imminent risk of extensive anterior wall STEMI [13]. Also, a stress test should be avoided as shown in a previous case who developed acute STEMI and cardiac arrest shortly after starting exercise [9].

Given that Wellens syndrome is associated with a large territory of jeopardized myocardium, clinical awareness of its ECG pattern is of paramount importance to allow timely coronary angiography and revascularization [1]. Care must be taken to avoid premature diagnostic closure, as a variety of cardiac and noncardiac conditions are also associated with precordial T wave inversion, such as Takotsubo cardiomyopathy, arrhythmogenic right ventricular dysplasia, hypertrophic cardiomyopathy, pulmonary embolism, and increased intracranial pressure [14].

## 6 Conclusion

Wellens syndrome is an urgent condition associated with imminent risk of acute extensive anterior wall STEMI. It can be diagnosed by appropriate clinical context and recognition of its ECG characteristics. Clinicians should not rely on cardiac biomarkers. In actively symptomatic patients, the ECG may not show the classic Wellens sign because of pseudonormalization. Clinical awareness is crucial to allow timely diagnosis and coronary revascularization.

## Author Contributions

**Juthipong Benjanuwattra:** writing – original draft, writing – review and editing. **Cristian Castillo‐Rodriguez:** writing – original draft. **Manisha Das:** conceptualization, supervision, visualization. **Shaheer Zulfiqar:** supervision, writing – review and editing. **Imran Arif:** supervision, validation, writing – review and editing.

## Consent

Written informed consent was obtained from the patient to publish this report in accordance with the journal's patient consent policy.

## Conflicts of Interest

The authors declare no conflicts of interest.

## Data Availability

The data that support the findings of this study are available upon request from the corresponding author. The data are not publicly available due to privacy or ethical restrictions.
